# Gene expression profiling in primary breast cancer distinguishes patients developing local recurrence after breast-conservation surgery, with or without postoperative radiotherapy

**DOI:** 10.1186/bcr1997

**Published:** 2008-04-22

**Authors:** Emma Niméus-Malmström, Morten Krogh, Per Malmström, Carina Strand, Irma Fredriksson, Per Karlsson, Bo Nordenskjöld, Olle Stål, Görel Östberg, Carsten Peterson, Mårten Fernö

**Affiliations:** 1 Institute of Clinical Sciences, Department of Oncology, University Hospital, SE 221 85 Lund, Sweden; 2 Computational Biology and Biological Physics, Department of Theoretical Physics, Lund University, SE 223 68 Lund, Sweden; 3 Department of Surgery, Karolinska University Hospital in Solna, SE 171 76 Stockholm, Sweden; 4 Department of Oncology, Sahlgrenska University Hospital, SE 413 45 Gothenburg, Sweden; 5 Department of Clinical and Experimental Medicine, Division of Oncology, Linköping University, University Hospital, SE 581 85 Linköping, Sweden; 6 Department of Pathology, Halmstad Hospital, SE 302 33 Halmstad, Sweden

## Abstract

**Introduction:**

Some patients with breast cancer develop local recurrence after breast-conservation surgery despite postoperative radiotherapy, whereas others remain free of local recurrence even in the absence of radiotherapy. As clinical parameters are insufficient for identifying these two groups of patients, we investigated whether gene expression profiling would add further information.

**Methods:**

We performed gene expression analysis (oligonucleotide arrays, 26,824 reporters) on 143 patients with lymph node-negative disease and tumor-free margins. A support vector machine was employed to build classifiers using leave-one-out cross-validation.

**Results:**

Within the estrogen receptor-positive (ER^+^) subgroup, the gene expression profile clearly distinguished patients with local recurrence after radiotherapy (n = 20) from those without local recurrence (n = 80 with or without radiotherapy). The receiver operating characteristic (ROC) area was 0.91, and 5,237 of 26,824 reporters had a *P *value of less than 0.001 (false discovery rate = 0.005). This gene expression profile provides substantially added value to conventional clinical markers (for example, age, histological grade, and tumor size) in predicting local recurrence despite radiotherapy. Within the ER^- ^subgroup, a weaker, but still significant, signal was found (ROC area = 0.74). The ROC area for distinguishing patients who develop local recurrence from those who remain local recurrence-free in the absence of radiotherapy was 0.66 (combined ER^+^/ER^-^).

**Conclusion:**

A highly distinct gene expression profile for patients developing local recurrence after breast-conservation surgery despite radiotherapy has been identified. If verified in further studies, this profile might be a most important tool in the decision making for surgery and adjuvant therapy.

## Introduction

The addition of postoperative radiotherapy to breast-conservation surgery in patients with lymph node-negative breast cancer has been shown to reduce the 10-year risk of local recurrence from 29.2% to 10% [1]. However, more than half of the patients will never develop local recurrence whether given radiotherapy or not and a small proportion of the patients will develop local recurrence despite being given radiotherapy. Besides tumor-involved margins, generally accepted risk factors for the development of local recurrence are young age and multicentricity [2-5]. A number of other risk factors have been reported (for example, extensive intraductal component [6], family history [7], lymphovascular invasion [2,8-10], lobular cancer [11], and estrogen receptor-negative (ER^-^) status [10]), but their clinical usefulness so far is limited. If the patients who develop local recurrence despite radiotherapy can be identified, other treatment strategies should be considered. No factor hitherto has been found to be clinically useful for the identification of patients developing local recurrence after radiotherapy.

Gene expression analyses have been found to be useful for molecular subclassification of breast cancer and also have shown promising results for predicting distant recurrence [12-17]. Concerning prediction of local recurrence, only a few studies have been reported. Cheng and colleagues [18] demonstrated two sets of gene expression profiles to be associated with local recurrence after mastectomy. Today, however, the majority of patients with breast cancer are operated on with breast-conservation surgery. As conventional risk factors for local recurrence after mastectomy differ from those after breast-conservation surgery, these findings may not be applicable when using less extensive surgery. Two recent studies included only patients treated with breast-conservation surgery: one was unable to find a distinguishing gene expression profile [19], whereas the other could only separate patients developing local recurrence after radiotherapy from patients not developing local recurrence by means of a predefined gene list, the wound-response signature [20]. This signature has been suggested to provide a possible link between cancer progression and wound healing and originally was defined as the fibroblast core serum response [21]. The material in the study by Nuyten and colleagues [20] was heterogeneous with regard to margin status, ER status, lymph node status, adjuvant systemic treatment (47% with and 53% without), and radiotherapy (including both standard and boost treatment). This heterogeneity might be the reason for not finding a significant gene profile in this study when using the whole set of genes. As far as the importance of considering ER status in gene expression analyses, today it is generally accepted that ER^+ ^and ER^- ^breast tumors have remarkably distinct gene expression profiles [22,23] and this subdivision of ER status has been successfully applied when predicting distant recurrence [14,24].

**Figure 1:**
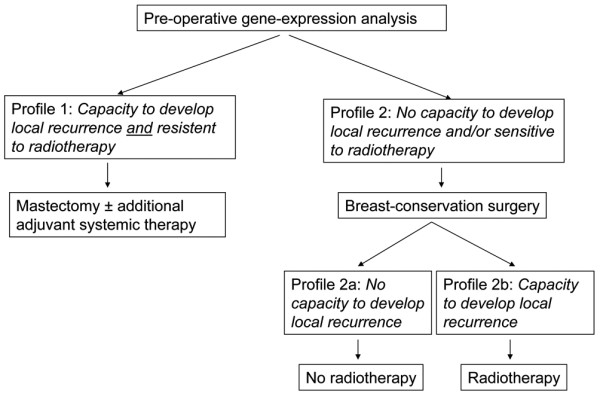
A hypothetical clinical routine scheme for the choice of surgery and radiotherapy after preoperative gene expression analysis.

Our study aimed at elucidating whether gene expression analysis is useful in predicting tumor sensitivity to radiotherapy and capacity to develop local recurrence in a patient material homogenous with regard to tumor-free margins, lymph node status, and radiotherapy (only standard doses). A predictive gene expression profile might impact the choice of both surgery and radiotherapy. A hypothetical clinical routine scheme, demonstrating three treatment options, is outlined in Figure 1. After a preoperative analysis of the gene expression profile, the first step is to identify the patients who will develop local recurrence despite radiotherapy. For this group, mastectomy might be a better choice. The second step is to separate those patients with no capacity to develop local recurrence and therefore not in need of radiotherapy after breast-conservation surgery from those with a capacity to develop local recurrence and in need of radiotherapy.

## Materials and methods

### Patients

#### Study design, inclusion criteria, and sample collection

Frozen tumor samples were collected from patients representing the following four groups: (a) LR^+^RT^+ ^(local recurrence developed after radiotherapy), (b) LR^-^RT^+ ^(no local recurrence after radiotherapy), (c) LR^+^RT^- ^(local recurrence developed, no radiotherapy given), and (d) LR^-^RT^- ^(no local recurrence, no radiotherapy given). All patients were operated on with breast-conservation surgery and axillary clearance with no lymph node involvement, tumor size of less than or equal to 30 mm (two patients had tumors measuring 32 and 40 mm, respectively, and one was T2 without any further information on size), tumor-free margins (>1 mm), no multicentricity, and with frozen tumor tissue with good RNA quality available. Local recurrence was defined as the appearance of a new breast tumor in the ipsilateral residual breast parenchyma in the overlying skin or in the scar. Patients with recurrence in the contralateral breast or with distant metastases prior or simultaneous to local recurrence were excluded. In the first inclusion, 102 patients from a randomized clinical trial in the South and West health care regions in Sweden [25] and 19 patients from a population-based cohort study with a nested case-control study (Stockholm and South Sweden) [3,26] were included (Tables 1 and 2). To perform gene expression profiling in a more homogenous material with regard to ER status and yet with a sufficient number of cases in all four subgroups, 22 additional ER^+ ^tumors from the South-East and South health care regions were included in a second inclusion (Tables 1 and 2). The study was approved by the Ethics Committee at Lund University (Lund, Sweden). Patient and primary tumor characteristics and follow-up information were collected from the patients' medical records.

**Table 1:** Clinical and pathological characteristics of the 77 patients receiving radiotherapy, with or without the development of local recurrence

All	LR^+^RT^+^		LR^-^RT^+^
Time to local recurrence, months	n = 30		n = 47

Median			
Range	50		-
Follow-up, months	8–149		-
Median			
Range	-		85
Tamoxifen	-		62–231
Chemotherapy	6		1
Tamoxifen and chemotherapy	0		0
No adjuvant treatment	0		0
	24		46

Inclusion 1 and 2	Inclusion 1	Inclusion 2	Inclusion 1

Menopause			
Pre	12	5	13
Post	7	1	33
Not available	0	5	1
Age at operation, years			
Median	48	47	57
Range	27–63	34–73	33–73
Size, millimeters			
Median	14	15	15
Range	2–32	10–20	4–22
Not available	0	2^a^	0
Grade			
1	3	3	22
2	8	6	13
3	7	1	10
Not available	1	1	2
Estrogen receptor			
Positive	9	11	42
Negative	10	0	5
Progesterone receptor			
Positive	5	9	31
Negative	13	2	11
Not available	1	0	5
Health care region			
South	8	2	30
West	2	0	9
South-East	0	9	0
Stockholm	9	0	8

^a^One T1 and one T2. LR^-^RT^+ ^= no local recurrence after radiotherapy; LR^+^RT^+ ^= local recurrence developed after radiotherapy.

**Table 2:** Clinical and pathological characteristics of the 66 patients, not receiving radiotherapy, with or without the development of local recurrence

All	LR^+^RT^-^	LR^-^RT^-^	
	n = 22	n = 44	

Time to local recurrence, months			
Median	35	-	
Range	11–96	-	
Follow-up, months			
Median	-	84	
Range	-	21–166	
Tamoxifen	2	4	
Chemotherapy	1	1	
Tamoxifen and chemotherapy	1	0	
No adjuvant treatment	18	39	

Inclusion 1 and 2	Inclusion 1	Inclusion 1	Inclusion 2

Menopause			
Pre	9	3	4
Post	13	30	2
Not available	0	0	5
Age at operation, years			
Median	53	61	49
Range	44–73	45–70	40–62
Size, millimeters			
Median	15	13	16
Range	7–30	6–40	10–26
Not available	0	0	0
Grade			
1	4	13	5
2	10	9	3
3	5	8	3
Not available	3	3	0
Estrogen receptor			
Positive	14	27	11
Negative	8	6	0
Progesterone receptor			
Positive	15	17	11
Negative	7	14	0
Not available	0	2	0
Health care region			
South	9	21	11
West	13	10	0
South-East	0	0	0
Stockholm	0	2	0

LR^-^RT^- ^= no local recurrence, no radiotherapy given; LR^+^RT^- ^= local recurrence developed, no radiotherapy given.

### Treatment

Postoperative radiotherapy with a median absorbed dose of 50 Gy (range 48 to 54 Gy) was given in 24 to 27 fractions in one series to the remaining breast parenchyma. Adjuvant systemic therapy was given to 16 patients (Tables 1 and 2).

### Conventional factors

Histological grade was re-evaluated according to Elston and Ellis [27]. ER and progesterone receptor content were analyzed routinely after primary operation with enzyme immunoassay according to kit instructions (Abbott Laboratories, Diagnostics Division, Chicago, IL, USA) and expressed as femtomoles per milligram of cytosol protein. Receptor values greater than or equal to 25 fmol/mg protein were considered positive.

### Gene expression analysis

RNA was extracted from freshly frozen invasive breast tumors as previously described [28]. The RNA quality was assessed using an Agilent 2100 Bioanalyzer (Agilent Technologies, Santa Clara, CA, USA), and the RNA integrity number (RIN) method [29] was used to validate the RNA quality. Twenty-one samples were excluded due to RIN values of below 6. Labeling and hybridization were performed as previously described [28]. By means of Human Genome Oligo Set Version 2.1 (containing 21,329 70-mer probes) and Human Genome Oligo Set Version 2.1 Upgrade 27 (containing 5,462 70-mer probes), oligonucleotide arrays were produced by the Swegene DNA Microarray Resource Centre, Lund University [30]. In inclusion step 1, probes were printed in duplicate, creating 55 K slides, and in inclusion step 2, in single, creating 27 K slides.

### Statistics

Wilcoxon rank sum tests, Sammon maps, and support vector machine (SVM) classifications [31] were performed with the statistical language R [32] using the libraries MASS (Sammon) and e1071 (SVM). For the SVM, only genes with no missing values were used. For the LR^+^RT^+ ^versus LR^-^RT^+^/LR^-^RT^- ^groups, the numbers of genes with no missing values were 9,128, 13,362, and 8,834 for the ER^+^, ER^-^, and combined ER groups, respectively. For the LR^+^RT^- ^versus LR^-^RT^- ^groups, they were 11,209, 13,547, and 10,658, respectively. For the wound-response genes, the corresponding numbers were 93, 120, and 91 for the LR^+^RT^+ ^versus LR^-^RT^+^/LR^-^RT^- ^groups and 105, 122, and 99 for the LR^+^RT^- ^versus LR^-^RT^- ^groups. Leave-one-out cross-validation was used. When a sample was left out, the SVM was trained on the remaining samples, and the distance to the maximal margin hyperplane (the decision value) was calculated for the left-out sample. A linear kernel was used and the cost of constraints violation (C constant) was fixed to one. No parameter tuning was performed even if the use of another layer of cross-validation might have improved the results. The goal of this study was to prove that gene expression profiles can distinguish the groups, not to find the optimal classifier. Actually, the optimal classifier does not even need to be an SVM. We also minimized potential suspicions about information leak by restricting the parameters of the SVM to the default values of the R function svm. A receiver operating characteristic (ROC) curve and area were calculated using the decision values. The expected average value of the ROC area is 0.5 if there is no discrimination between the groups. Due to random variations, ROC areas above 0.5 are often obtained even when there is no discrimination between the groups. To distinguish a real discrimination between the groups from the case of no discrimination, a p-value was calculated. A small p-value makes it unlikely that the ROC area can be reconciled with the case of no discrimination. The p-value was calculated by a permutation test. The local recurrence labels were shuffled randomly 1,000 times and the ROC areas were found for the corresponding classifications. The *P *value was calculated as the fraction of the 1,000 permutations that had an ROC area larger than the real one. If the *P *value was zero, the random ROC areas were fitted to a normal distribution and the area under the tail above the real ROC area was used as the *P *value. The *P *value of the ROC area for the case of a fixed test set (that is, no cross-validation) was calculated by a permutation test of the labels in the test set. Odds ratios, confidence intervals of odds ratios, and *P *values of odds ratios were calculated with the R function Fisher test, which uses the conditional maximum likelihood estimator.

### Gene Ontology

The Gene Ontology (GO) [33] OBO (open biomedical ontologies) file of 14 November 2006 was used. Gene annotation was performed using ACID (Array Clone Information Database), which is a publicly available web application that provides GO categories for genes [34]. A total of 6,841 GO categories belonging to 'Cellular component', 'Biological process', or 'Molecular function' had at least one gene in common with our data. The genes were ranked according to their Wilcoxon rank sum *P *value between LR^+^RT^+ ^and LR^-^RT^+^/LR^-^RT^- ^groups in the ER^+ ^group. A Wilcoxon rank sum test was performed for each GO category to test for over-representation of genes toward the top of the ranked gene list using Catmap [35].

## Results

### Patients with a capacity to develop local recurrence despite radiotherapy

To identify this group of patients, we compared LR^+^RT^+ ^versus LR^-^RT^+^/LR^-^RT^- ^groups. There was an association between the LR^+^RT^+ ^group and ER^- ^status (odds ratio 6.8, 95% confidence interval 2.0 to 24; *P *= 0.0007) (Table 3). In this analysis, only the patients from the first inclusion were used, as the second inclusion was made on ER and local recurrence status. Age can also distinguish the LR^+^RT^+ ^group. Histological grade was marginally able to separate the LR^+^RT^+ ^group from the LR^-^RT^+^/LR^-^RT^- ^group, whereas tumor size was not (Table 3).

**Table 3:** A comparison between the LR^+^RT^+ ^and LR^-^RT^+^/LR^-^RT^- ^subgroups

Factor	LR^+^RT^+^	LR^-^RT^+^/LR^-^RT^-^	*P *value
ER status, number			
Negative	10	11	
Positive	9	69	0.0007^a^
Median age, years			
All	48	61	0.00004^b^
ER^- ^subgroup	49.5	53	0.12
ER^+ ^subgroup	46	61	0.002
Histological grade, number			
1	3	35	
2	8	22	
3	7	18	0.055^a^
Median tumor size, millimeters	15	15	0.95^b^

Only cases from inclusion 1 are included. ^a^Fisher exact test. ^b^Wilcoxon rank sum test. ER = estrogen receptor; LR^-^RT^- ^= no local recurrence, no radiotherapy given; LR^-^RT^+ ^= no local recurrence after radiotherapy; LR^+^RT^+ ^= local recurrence developed after radiotherapy.

**Figure 2:**
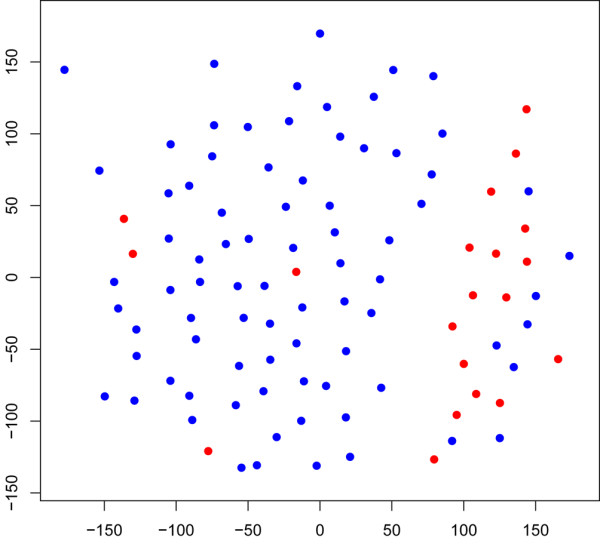
A Sammon map of the 100 estrogen receptor-positive patients within the LR^+^RT^+ ^group (red dots, 20 patients) and the LR^-^RT^+^/LR^-^RT^- ^group (blue dots, 80 patients). The Sammon map was performed with all 26,824 reporters. Euclidean distance in log_2 _expression values was used as the distance measure. LR^-^RT^- ^= no local recurrence, no radiotherapy given; LR^-^RT^+ ^= no local recurrence after radiotherapy; LR^+^RT^+ ^= local recurrence developed after radiotherapy.

**Figure 3:**
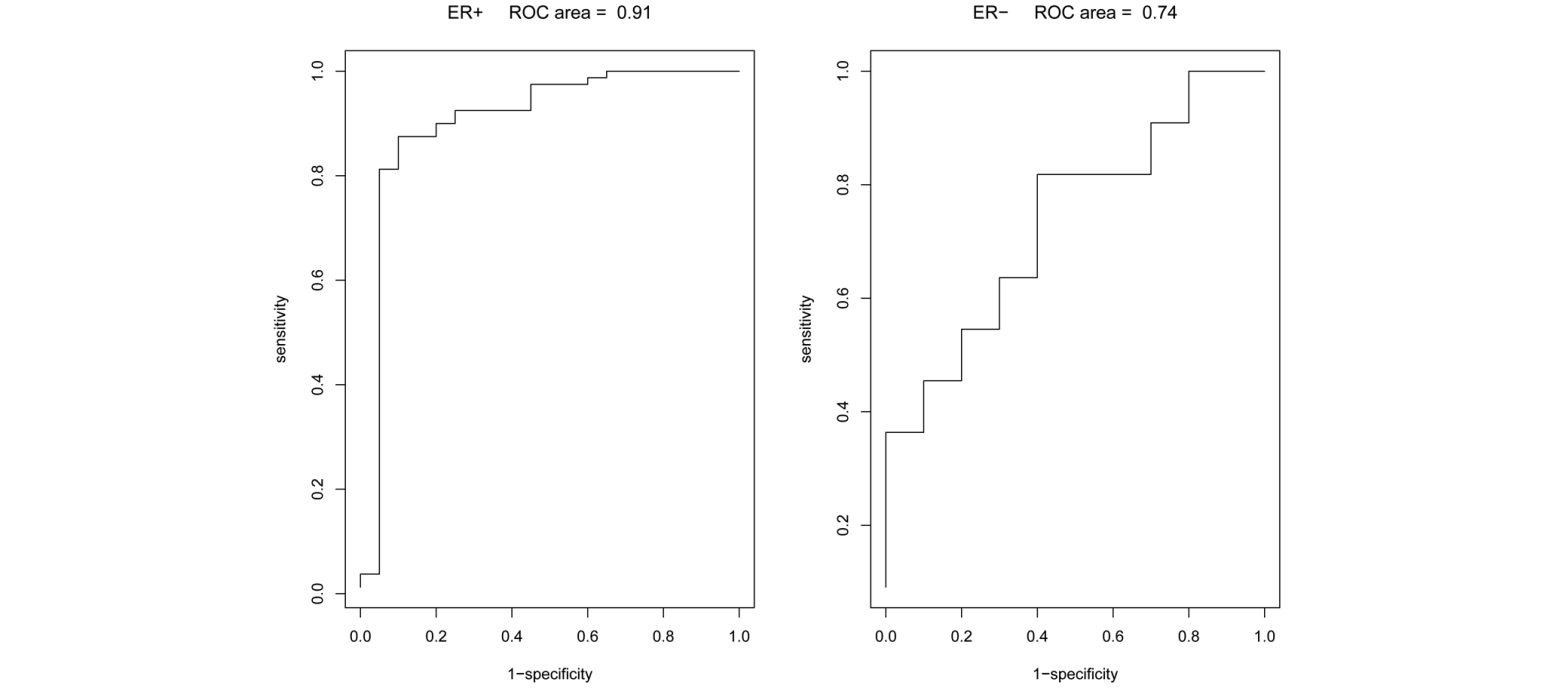
Receiver operating characteristic (ROC) curves for the support vector machine classification of LR^+^RT^+ ^versus LR^-^RT^+^/LR^-^RT^- ^groups within the estrogen receptor-positive (ER^+^) group (left part) and estrogen receptor-negative (ER^-^) group (right part). The specificity is defined as the fraction of the LR^-^RT^+^/LR^-^RT^- ^patients correctly classified, and the sensitivity as the fraction of the LR^+^RT^+ ^patients correctly classified. LR^-^RT^- ^= no local recurrence, no radiotherapy given; LR^-^RT^+ ^= no local recurrence after radiotherapy; LR^+^RT^+ ^= local recurrence developed after radiotherapy.

After filtering the microarray data on low-quality spots and missing values, 26,824 reporters remained, representing 16,895 unique genes. From a Sammon map of the gene expression profiles of the 100 ER^+ ^patients from both inclusions, it was evident that the LR^+^RT^+ ^and LR^-^RT^+^/LR^-^RT^- ^groups were well separated even without gene selection (Figure 2). For supervised classification, an SVM was used. The areas under the receiver operating curve (ROC areas) were 0.91 (*P *= 9 × 10^-6^) within the ER^+ ^group, 0.74 (*P *= 0.08) within the ER^- ^group (Figure 3), and 0.83 (*P *= 9 × 10^-5^) within the combined ER^+^/ER^- ^group (data not shown). The ER^+ ^group was by far the larger group, which could explain the superior performance of the ER^+ ^group compared with the ER^- ^one. Also, the classification performance was deteriorated by combining ER^+ ^and ER^- ^in one SVM; it was preferable to use distinct SVMs for the two subpopulations (Figure 3). For the ER^+ ^group, at 90% sensitivity (18 of 20 LR^+^RT^+ ^correctly classified), the specificity was 87.5% (70 of 80 LR^-^RT^+^/LR^-^RT^- ^correctly classified), and at 90% specificity (72 of 80 LR^-^RT^+^/LR^-^RT^- ^correctly classified), the sensitivity was 80% (16 of 20 LR^+^RT^+ ^correctly classified).

As age is a risk factor for local recurrence, we investigated whether the gene expression profiling has classification ability beyond that of age in the ER^+ ^subgroup. We constructed a training set from the 77 patients who were either older than 50 years and in the LR^-^RT^+^/LR^-^RT^- ^group or younger than 50 years and in the LR^+^RT^+ ^group. The test set consisted of the remaining 23 patients (for example, those who were either younger than 50 years and in the LR^-^RT^+^/LR^-^RT^- ^group or older than 50 years and in the LR^+^RT^+ ^group). The point is that the test set chosen contains patients who behave exactly opposite to the usual connection between age and local recurrence. Applying an SVM, we obtained an ROC area of 0.88 (*P *= 0.002). Furthermore, we checked the influence of health care regions by using the 68 samples from the South and South-East health care regions as a training set and the 32 samples from the West or Stockholm health care regions as a test set. The specific split into health care regions was done to get a reasonable amount of samples in LR^+^RT^+ ^and LR^-^RT^+^/LR^-^RT^- ^groups in both the training and the test set. No optimizations were performed in this regard. The ROC area of 0.87 (*P *= 0.002) shows that the classifier indeed works across health care regions.

The wound-response signature genes, also known as the core serum response genes [21], were shown to have the ability of partially predicting local recurrence [20]. We mapped the wound-response signature to our microarrays and performed an SVM classification using only this signature. The ROC areas were 0.75 (*P *= 0.007) within the ER^+ ^group, 0.75 (*P *= 0.08) within the ER^- ^group, and 0.61 (*P *= 0.10) within the combined ER^+^/ER^- ^group.

### Differentially expressed genes

**Figure 4:**
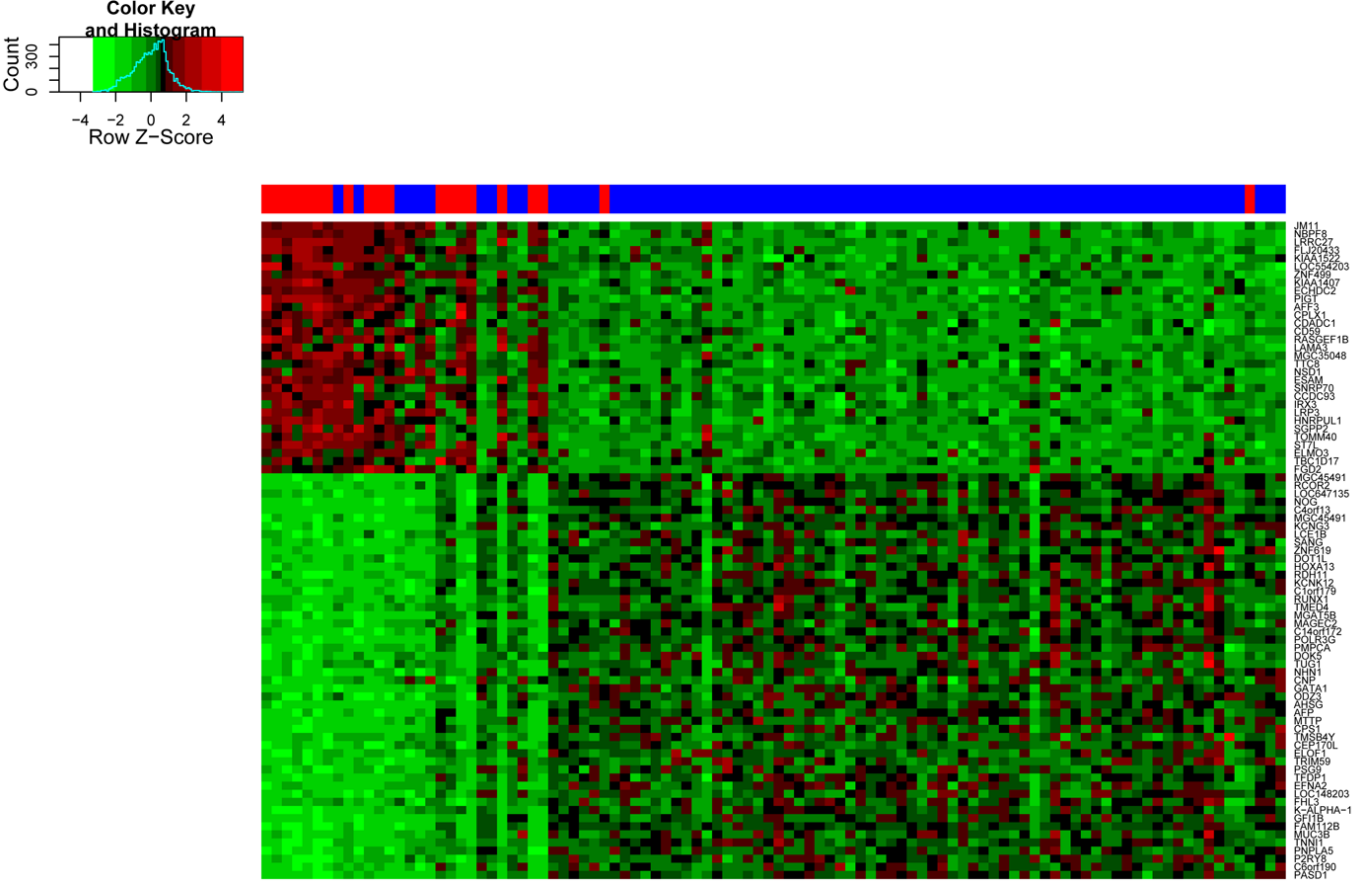
The 81 most important genes. A heatmap of 81 genes (rows) and 100 patients (columns) with patients ordered according to their leave-one-out support vector machine classification value: estrogen receptor-positive patients within LR^+^RT^+ ^(red, n = 20) and LR^-^RT^+^/LR^-^RT^- ^(blue, n = 80) groups. The gene selection and ordering are described in the text. Expression values are logarithmically transformed, centered around zero, and normalized to unit standard deviation. LR^-^RT^- ^= no local recurrence, no radiotherapy given; LR^-^RT^+ ^= no local recurrence after radiotherapy; LR^+^RT^+ ^= local recurrence developed after radiotherapy.

A Wilcoxon rank sum test between LR^+^RT^+ ^and LR^-^RT^+^/LR^-^RT^- ^groups within the ER^+ ^subgroup was performed for all 26,824 reporters. A clear over-representation of genes with small *P *values was found; for example, there are 5,237 reporters with a *P *value below 0.001 corresponding to a Benjamini-Hochberg [36] false discovery rate of 0.005. A heatmap of the 81 genes with a known gene name, no missing values, and a Wilcoxon rank sum test *P *value between the LR^+^RT^+ ^and LR^-^RT^+^/LR^-^RT^- ^groups of below 10^-6 ^is shown in Figure 4. A GO analysis was performed using Catmap [35]. A total of 6,841 GO categories belonging to 'Cellular component', 'Biological process', or 'Molecular function' were included. At a false discovery rate of 0.05, only the four categories of cytosolic ribosome (sensu Eukaryota), eukaryotic 43S preinitiation complex, eukaryotic 48S initiation complex, and cytosolic small ribosomal subunit (sensu Eukaryota) were significant.

### Patients with no capacity to develop local recurrence

To identify this group of patients, we analyzed LR^+^RT^- ^versus LR^-^RT^- ^groups. ER^- ^status was weakly correlated with the LR^+^RT^- ^group (odds ratio 2.5, 95% confidence interval 0.6 to 11; *P *= 0.21) (Table 4). Young age was correlated with local recurrence in the ER^+ ^group (Table 4). Neither histological grade nor tumor size had the power to separate the two groups.

**Table 4:** A comparison between the LR^+^RT^- ^and LR^-^RT^- ^subgroups

Factor	LR^+^RT^-^	LR^-^RT^-^	*P *value
ER status, number			
Negative	8	6	
Positive	14	27	0.21^a^
Median age, years			
All	53	61	0.02^b^
ER^- ^subgroup	59.5	54	0.90
ER^+ ^subgroup	50.5	62	0.02
Histological grade, number			
1	4	13	
2	10	9	
3	5	8	0.19^a^
Median tumor size, millimeters	15	14	0.67^b^

Only cases from inclusion 1 are included. ^a^Fisher exact test. ^b^Wilcoxon rank sum test. ER, = estrogen receptor; LR^-^RT^- ^= no local recurrence, no radiotherapy given; LR^+^RT^- ^= local recurrence developed, no radiotherapy given.

An SVM gene expression classifier yielded an ROC area of 0.66 (*P *= 0.04) within the combined ER^+^/ER^- ^group. The ER^- ^and ER^+ ^subgroups were too small to give a significant result on their own, even though there was a tendency of discriminative power within the ER+ subgroup. (ROC area = 0.62; *P *= 0.14). For the wound-response signature, the ROC areas were 0.64 (*P *= 0.10) in the ER^+ ^group, 0.69 (*P *= 0.27) in the ER^- ^group, and 0.68 (*P *= 0.03) in the combined group.

## Discussion

We have found a highly significant gene expression profile associated with the development of local recurrence after breast-conservation surgery despite postoperative radiotherapy. If patients resistant to radiotherapy can be identified, they should be candidates for alternative treatment strategies such as mastectomy, other adjuvant treatments, and/or higher radiation doses as local recurrence implies an increased risk of both distant metastases and mortality [37-39]. So far, there are no markers useful in the clinic for the identification of radio-resistant breast cancer. We found both age and ER status to be associated with local recurrence after radiotherapy. However, our gene expression signature provides substantially added value to these factors and also to histological grade and tumor size. A hybrid classifier of age and gene expression should perform even better than age or gene expression alone. Due to the sample size in this study, we did not have the possibility to build such a hybrid classifier. We have focused on the question of whether gene expression analysis *per se *is useful for the identification of patients with different risks of developing local recurrences. A thorough and more specific evaluation of the gene list should be performed after a confirmative study in which not only the genes, but also the pathways in which they are involved, are considered. The high proportion of ribosomal-related genes is noteworthy but also needs to be confirmed. The samples were collected from four health care regions with different routines for handling fresh tumor tissue prior to freezing. However, we could clearly demonstrate that these differences did not influence the importance of the gene expression signature.

To our knowledge, only one previous study has reported a gene expression signature significantly associated with local recurrence after breast-conservation surgery [20], but only when using a predefined wound-response signature gene list. One reason for not finding a significant profile when using the entire gene set may be that their material, which included 17 local recurrences, was more heterogeneous than ours with regard to tumor-free margins, tumor size, lymph node status, and dose of radiotherapy. Furthermore, they did not separate the samples with regard to ER status. ER^+ ^and ER^- ^breast tumors are known to have distinct gene expression profiles and indeed we found a stronger gene expression profile when including only ER^+ ^tumors compared with when ER^- ^tumors were included (ROC area 0.91 compared with 0.83). This finding further strengthened the notion that ER^+ ^and ER^- ^breast cancer should be handled as two separate entities when evaluating gene expression data, as has previously been stated by authors in analyses of gene expression profiles associated with distant metastases [14,24,40]. In our material, the wound-response signature genes were able to predict local recurrence within both the ER^+ ^group and ER^- ^group with reasonable accuracy, whereas the prediction in the combined group was rather weak. For the ER^+ ^group and the combined group, the classification performance is inferior to the results obtained with all genes. This degradation of performance shows that the advantage of restricting the gene set used in the classifier to the focused set of wound-response signature genes, which are known to be relevant to cancer, is outweighed by the loss of information of discarding the majority of the genes. The reason that the SVM using all genes was so much better at classifying the combined group than the wound-response signature genes is probably that the ER signal is contained in the full gene set but is more or less absent in the wound-response signature genes. With respect to individual samples, it is seen that the samples that were misclassified with all genes were also misclassified with the wound-response signature genes but that many of the misclassified samples with the wound-response signature genes were correctly classified with all genes.

For the identification of patients with no capacity to develop local recurrence, we compared the LR^+^RT^- ^and LR^-^RT^- ^subgroups. The gene expression signal was weaker, but still significant (ROC area = 0.66). The reason for a weaker signal may be the small number of patients (n = 66). For the wound-response genes, there is a tendency for correct predictions, but the results are too weak to draw any conclusions. More samples would be needed to test it further.

Today, the vast majority of breast cancer patients are treated with radiotherapy after breast-conservation surgery to lower the risk of local recurrence. However, a large proportion of these patients have a very low risk for local recurrence, and the positive effects must be weighed against costs and side effects of the treatment. The identification of patients with a very low risk to develop local recurrence, and consequently not in need of postoperative radiotherapy, is of great clinical importance.

No patient included in our study had tumor-involved margins, a risk factor for local recurrence. The local recurrence rate is influenced by the amount of uninvolved breast parenchyma surrounding the tumor as higher recurrence rates have been reported after lumpectomy [41] than after sector resection [25] or quadrantectomy [42]. With smaller resection margins, microscopic residual tumor is more likely to be present in the breast and the administered radiotherapy dose may be too low to give complete protection for local recurrence. Thus, it cannot be excluded that the gene expression profile can be influenced by resection margins.

Our gene expression profile was associated with local recurrence after radiotherapy at commonly used doses. Higher doses of radiation, with a boost of 16 Gy, have been shown to significantly reduce the local recurrence rate, particularly in patients below 50 years of age [43]. Unfortunately, higher radiation doses cause a less satisfactory cosmetic result [44]. Whether gene profiling also can be used for identification of patients in need of a boost is unclear at present.

One might speculate why the gene expression profile method performs significantly better for the prediction of local recurrence than for the prediction of distant metastasis. It is possible that the gene profile associated with the development of local recurrence despite radiotherapy (indicating radio-resistance) is more homogeneous than the one associated with distant recurrences. It is believed that the development of metastases is a more complicated process and that different groups of genes may be of variable importance in distinguished subgroups of breast cancer.

## Conclusion

We have found a very promising gene expression profile for predicting local recurrence despite radiotherapy – a profile that might be associated with radio-resistance. The signature provides substantially added value to the conventional factors used to predict risk of local recurrence. If confirmed in further studies, this profile might be a most important tool in the decision making for type of surgery and adjuvant therapy.

## Abbreviations

ER = estrogen receptor; ER^- ^= estrogen receptor-negative; ER^+ ^= estrogen receptor-positive; GO = Gene Ontology; LR^-^RT^- ^= no local recurrence, no radiotherapy given; LR^-^RT^+ ^= no local recurrence after radiotherapy; LR^+^RT^- ^= local recurrence developed, no radiotherapy given; LR^+^RT^+ ^= local recurrence developed after radiotherapy; RIN = RNA integrity number; ROC = receiver operating characteristic; SVM = support vector machine.

## Competing interests

The authors declare that they have no competing interests.

## Authors' contributions

EN-M participated in conceiving the design of the study, collecting the patient material and information of basic patient and tumor characteristics and clinical follow-up, performing gene expression and statistical analyses, interpreting data, and writing the paper. MK and CP participated in conceiving the design of the study, performing gene expression and statistical analyses, interpreting data, and writing the paper. EN-M and MK contributed equally to this manuscript. PM participated in conceiving the design of the study, collecting the patient material and information of basic patient and tumor characteristics and clinical follow-up, interpreting data, and writing the paper. CS participated in performing gene expression and statistical analyses, interpreting data, and writing the paper. IF, PK, BN, and OS participated in collecting the patient material and information of basic patient and tumor characteristics and clinical follow-up. GÖ re-evaluated the histopathological parameters. MF participated in conceiving the design of the study, interpreting data, and writing the paper. All authors read and approved the final manuscript.
